# Supplementary material for: MYCN Amplification, along with Wild-Type RB1 Expression, Enhances CDK4/6 Inhibitors’ Efficacy in Neuroblastoma Cells
Source: Int J Mol Sci. 2023 Mar 12;24(6):5408. doi: 10.3390/ijms24065408 (PMC10049239; doi:10.3390/ijms24065408)
Supplement: Supplementary file 1 [file ijms-24-05408-s001.zip › ijms-2146754-supplementary.pdf]

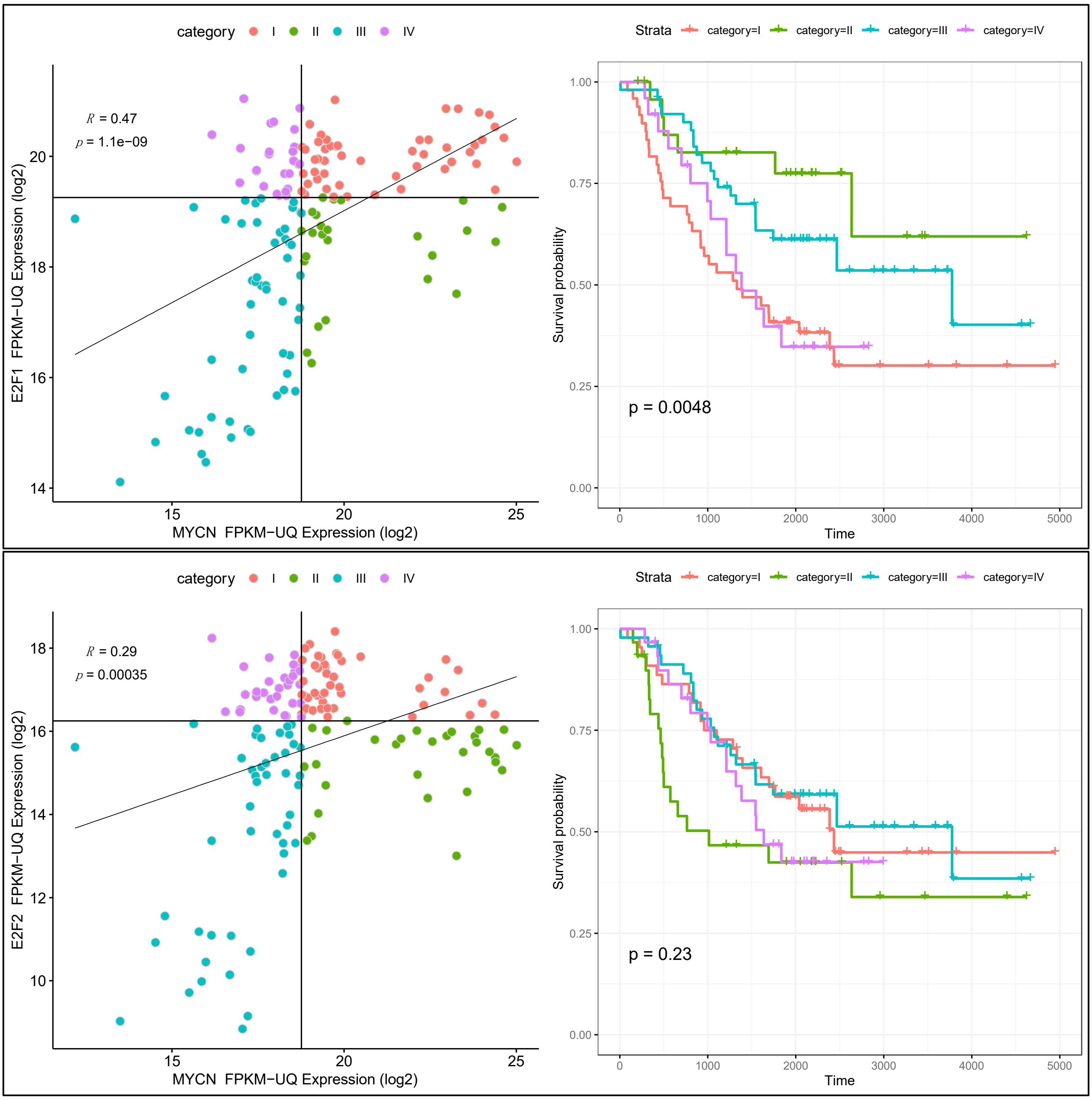

Supplementary Figure S1: Top Left: Spearman's correlation between E2F1 (y-axis) and MYCN (x-axis) in a cohort of 152 TARGET NBL patients. **Bottom Left**: Spearman's correlation between E2F2 (y-axis) and MYCN (x-axis) in a cohort of 152 TARGET NBL patients. Correlation coefficient (R) and p-value of correlation test are indicated.**Top/bottom Right**: Overall survival analysis of the TARGET NB dataset for each previously defined category. Correlation coefficient (R) and p-value of correlation test are indicated. Samples are divided in four categories (colored as in legend) according to median expression levels of E2F1/E2F2 and MYCN. Colors of Kaplan Meier curves as in legend. The p-value of the log-rank test is indicated. P-value of proportional hazard assumption was 0.34 and 0.073, respectively.

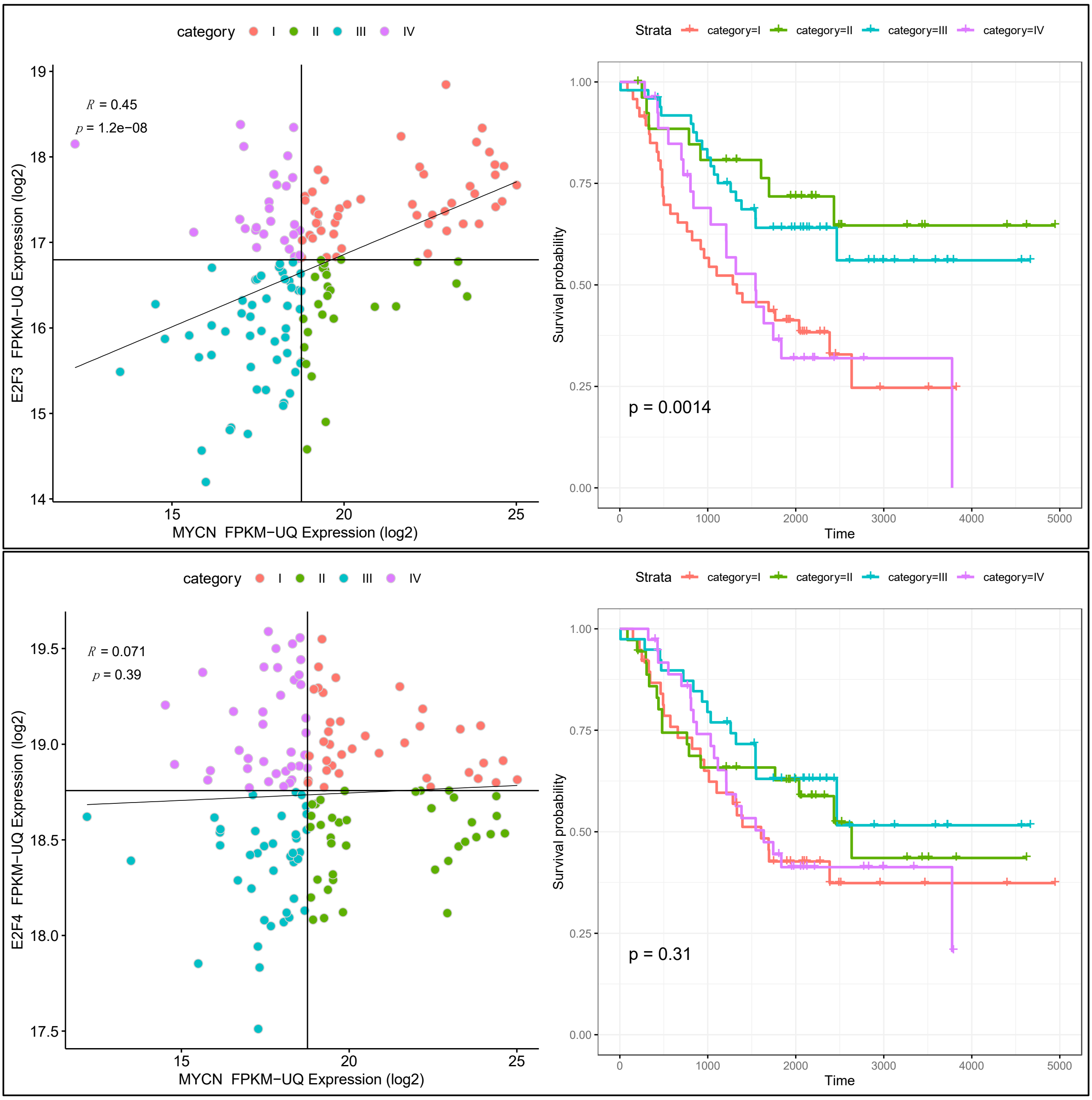

Supplementary Figure S2: Top Left: Spearman's correlation between E2F3 (y-axis) and MYCN (x-axis) in a cohort of 152 TARGET NBL patients. **Bottom Left**: Spearman's correlation between E2F4 (y-axis) and MYCN (x-axis) in a cohort of 152 TARGET NBL patients. Correlation coefficient (R) and p-value of correlation test are indicated. **Top/bottom Right**: Overall survival analysis of the TARGET NB dataset for each previously defined category. Correlation coefficient (R) and p-value of correlation test are indicated. Samples are divided in four categories (colored as in legend) according to median expression levels of E2F1/E2F2 and MYCN. Colors of Kaplan Meier curves as in legend. The p-value of the log-rank test is indicated. P-value of proportional hazard assumption was 0.27 and 0.22, respectively.

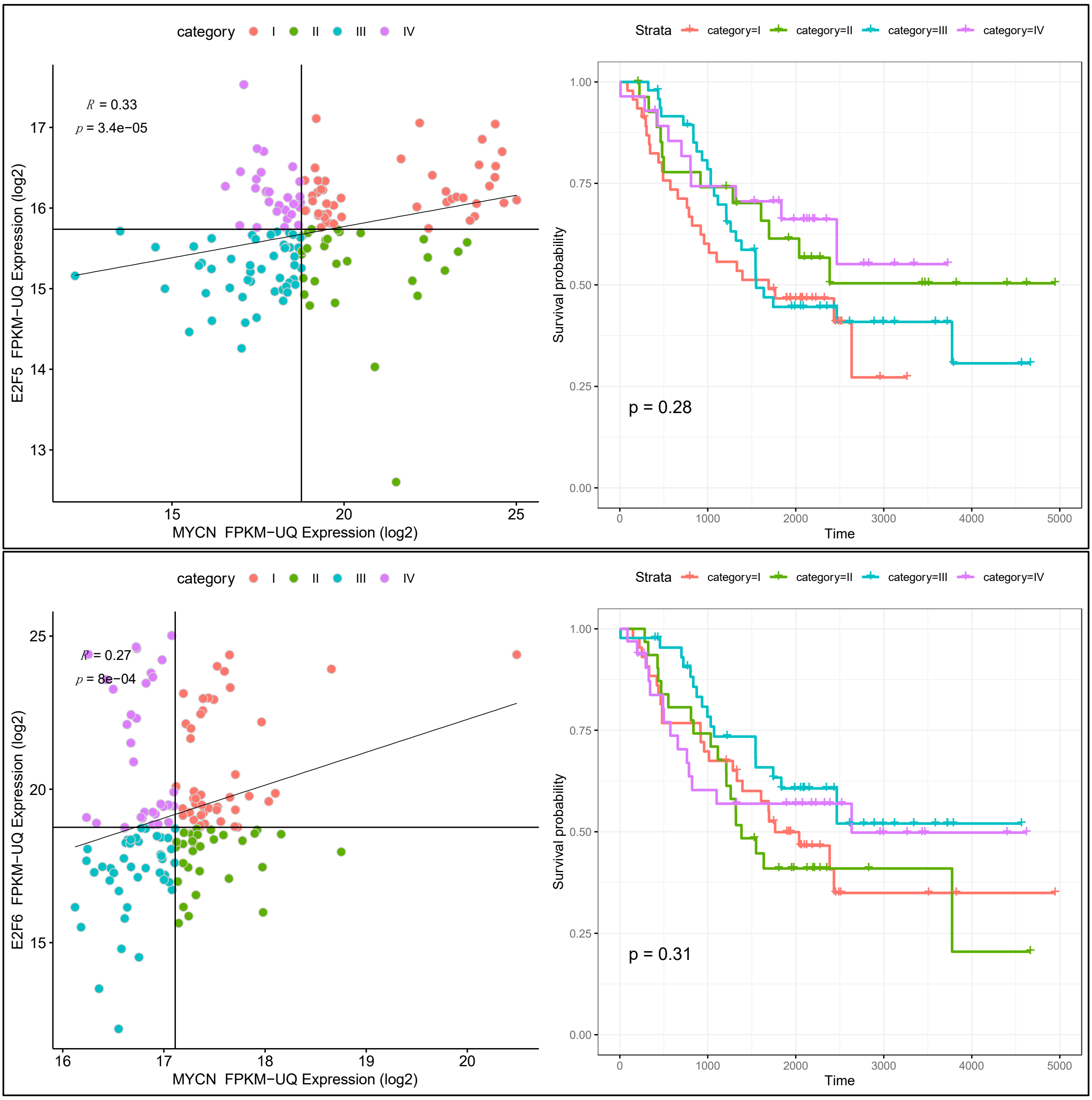

Supplementary Figure S3: Top Left: Spearman's correlation between E2F5 (y-axis) and MYCN (x-axis) in a cohort of 152 TARGET NBL patients. **Bottom Left**: Spearman's correlation between E2F6 (y-axis) and MYCN (x-axis) in a cohort of 152 TARGET NBL patients. Correlation coefficient (R) and p-value of correlation test are indicated. **Top/bottom Right**: Overall survival analysis of the TARGET NB dataset for each previously defined category. Correlation coefficient (R) and p-value of correlation test are indicated. Samples are divided in four categories (colored as in legend) according to median expression levels of E2F1/E2F2 and MYCN. Colors of Kaplan Meier curves as in legend. The p-value of the log-rank test is indicated. P-value of proportional hazard assumption was 0.15 and 0.064, respectively.

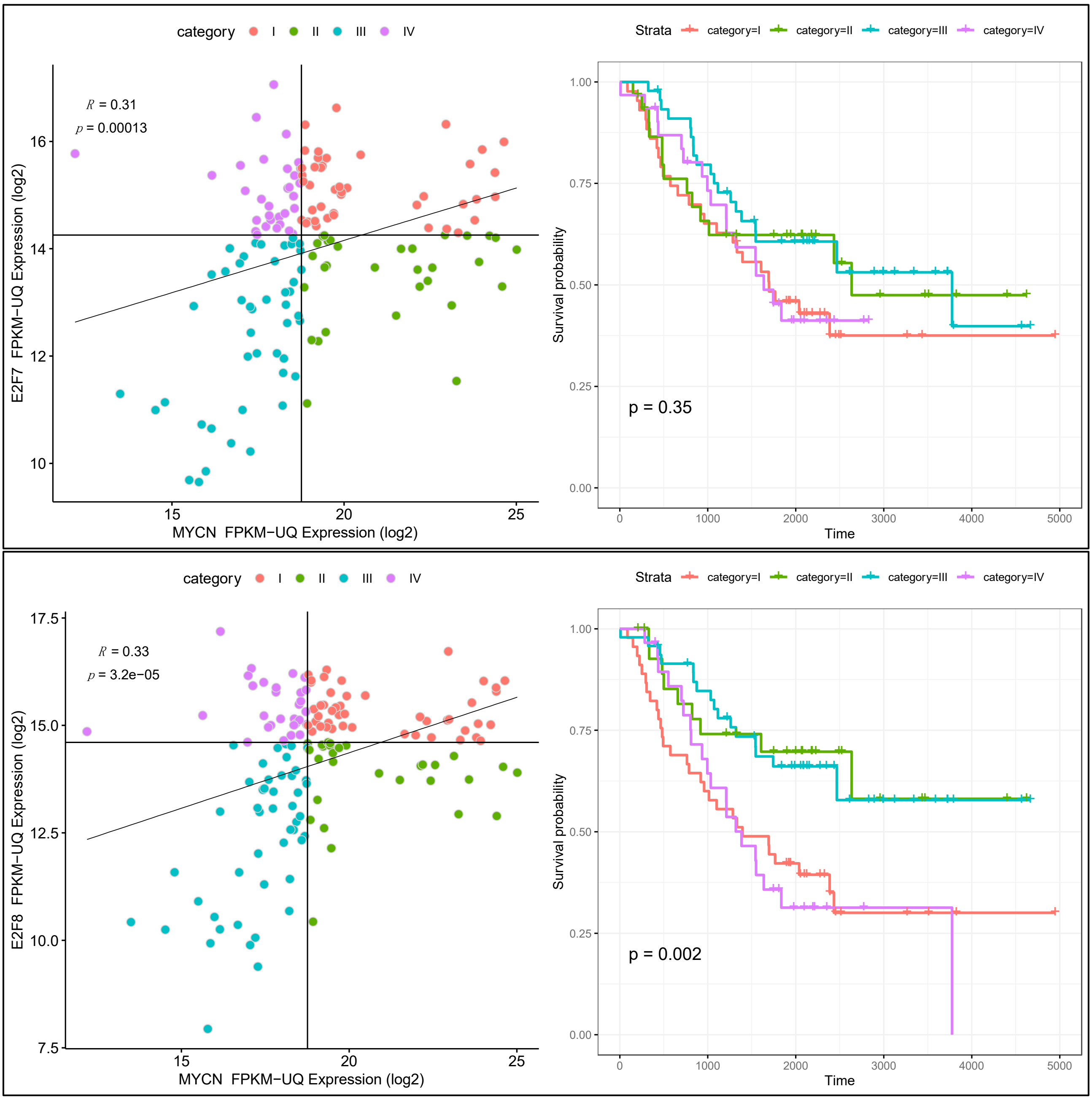

Supplementary Figure S4: Top Left: Spearman's correlation between E2F7 (y-axis) and MYCN (x-axis) in a cohort of 152 TARGET NBL patients. **Bottom Left**: Spearman's correlation between E2F8 (y-axis) and MYCN (x-axis) in a cohort of 152 TARGET NBL patients. Correlation coefficient (R) and p-value of correlation test are indicated. **Top/bottom Right**: Overall survival analysis of the TARGET NB dataset for each previously defined category. Correlation coefficient (R) and p-value of correlation test are indicated. Samples are divided in four categories (colored as in legend) according to median expression levels of E2F1/E2F2 and MYCN. Colors of Kaplan Meier curves as in legend. The p-value of the log-rank test is indicated. P-value of proportional hazard assumption was 0.23 and 0.19, respectively.

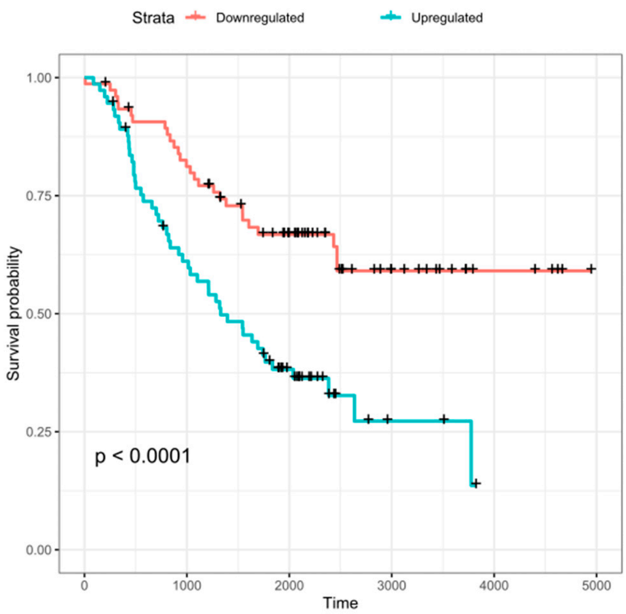

Supplementary Figure S5: Overall survival analysis of E2F3 expression in the TARGET NB dataset. Samples are divided in two categories according to median expression levels of E2F3 (Downregulated if below of the median of all samples, Upregulated if above the median). Colors of Kaplan Meier curves as in legend. The p-value of the log-rank test is indicated. P-value of proportional hazard assumption was 0.77.

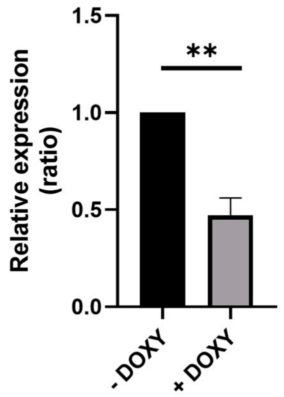

Supplementary Figure S6: conditional KD of RB1 in SKNAS cells through CRISPRi. Bars indicates average band intensity relative to tubulin of three (N=3) independent experiments. Error bars represent standard deviations. Two-tailed t-test was performed (** indicates p<0.005)

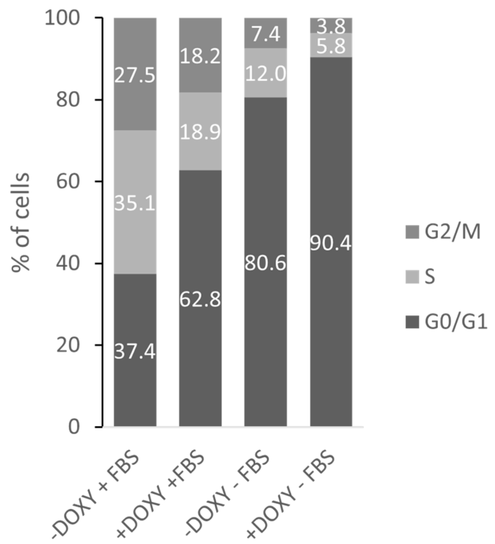

Supplementary Figure S7: Percentage of cells in each cell cycle phase as function of doxycycline and FBS

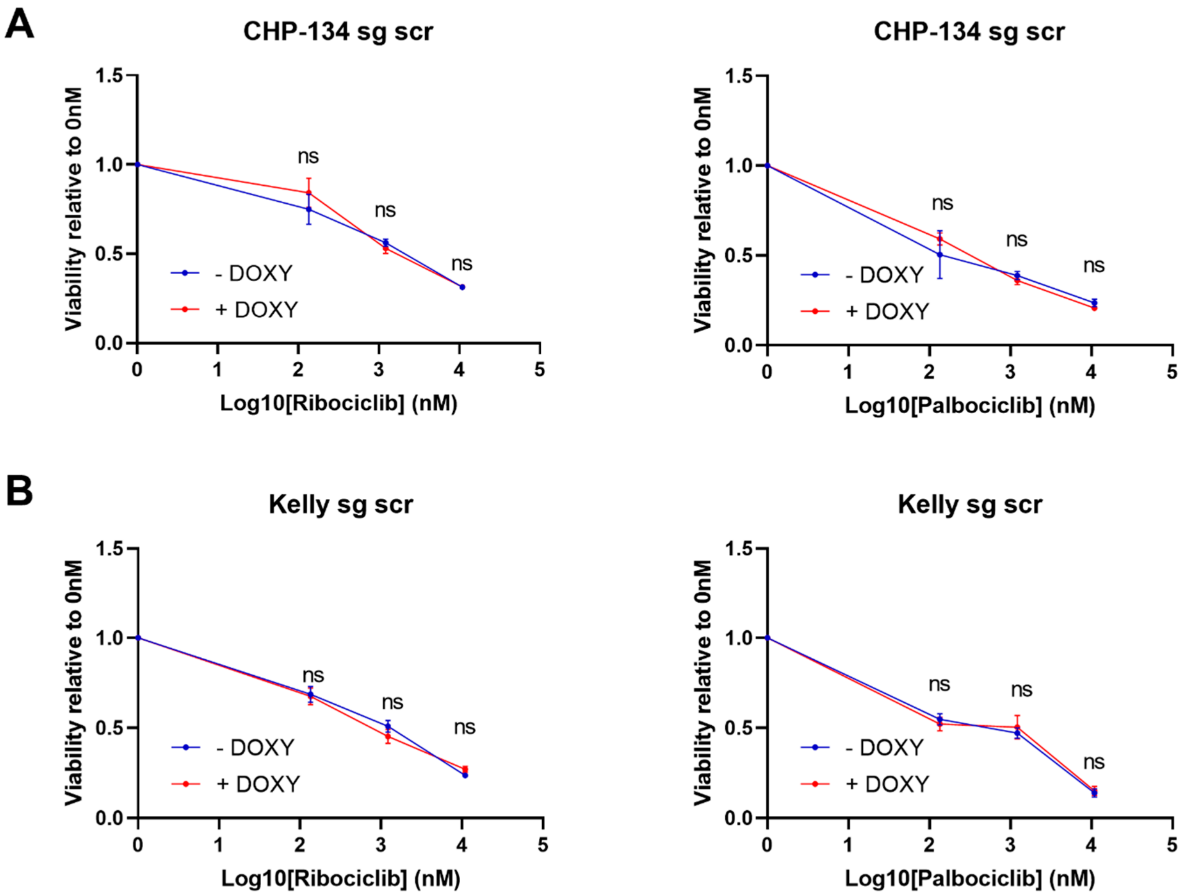

Supplementary Figure S8: CHP-124 and KELLY sg scr cells were seeded at low confluence and treated with ribociclib or palbociclib for 72 hours in the presence or absence of doxycycline. (A) MTS results of CHP-134 sg scr. (B) MTS results of KELLY sg RB. Data are plotted as absorbances normalized on the 0nM sample as averages of three (N=3) independent experiments. Error bars represented SD. Statistical analysis was performed by Two-way ANOVA comparisons between - doxy and + doxy samples per each concentration. ns indicated P > 0.05.

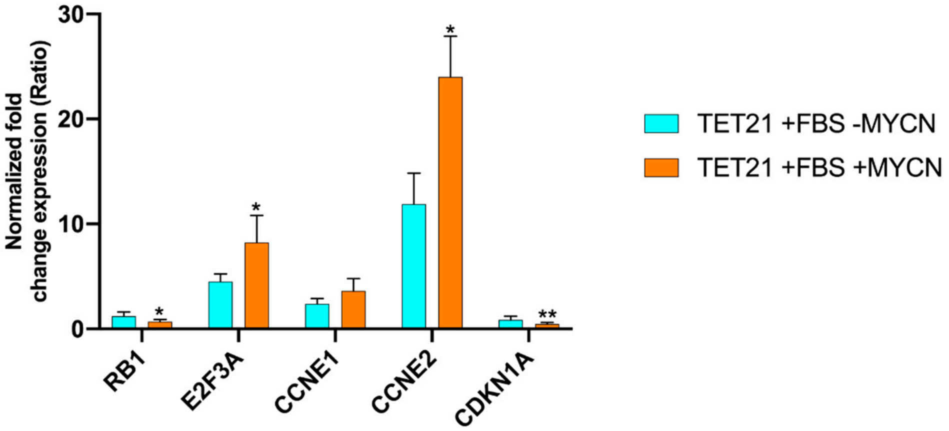

Supplementary Figure S9: (**C**) qRT-PCR TET21/N cells in the presence (-MYCN) or absence (+MYCN) of doxycycline. Data were normalized using the TBP housekeeping gene. The experiment was performed in duplicate (N=3). Data were plotted as the mean ± SD. Statistical analyses were performed using Two-way ANOVA. Error bars represent SD. *, ** indicated P < 0.05, 0.005, respectively.
